# The Use of Nerve Conduction Study to Evaluate the Effects of Frozen Sock Treatment on Docetaxel-Induced Peripheral Neuropathy in Breast Cancer Patients: A Prospective Clinical Trial

**DOI:** 10.3390/jcm14030864

**Published:** 2025-01-28

**Authors:** Eun-Young Kim, Mi-Yeon Lee, Bum-Chun Suh

**Affiliations:** 1 Department of Surgery, Kangbuk Samsung Hospital, School of Medicine, Sungkyunkwan University, Seoul 03181, Republic of Korea; gimo25@hanmail.net; 2 Division of Biostatistics, Department of Academic Research, Kangbuk Samsung Hospital, Seoul 03181, Republic of Korea; my7713.lee@samsung.com; 3 Department of Neurology, Kangbuk Samsung Hospital, School of Medicine, Sungkyunkwan University, Seoul 03181, Republic of Korea

**Keywords:** breast neoplasms, chemotherapy, docetaxel, peripheral neuropathy

## Abstract

**Background/Objectives**: Docetaxel is a cytotoxic agent for the treatment of breast cancer, and its toxicities include peripheral neuropathy (PN). This study evaluated the ability of frozen sock (FS) treatment to prevent docetaxel-induced PN by performing nerve conduction study (NCS). **Methods**: From October 2017 to October 2018, 48 patients who had invasive carcinoma and were planned for docetaxel treatment every three weeks were evaluated. Patients wore a FS on the right foot, and the left foot was not protected by the FS during docetaxel infusion. Motor and sensory NCS as well as nail and skin toxicities were assessed. **Results**: The amplitude and velocity of the motor and sensory nerves significantly decreased after three months in both feet. Before and after three months of chemotherapy, the compound motor action potentials (CMAPs) for the right peroneal nerve were 7.64 ± 2.42 and 6.81 ± 2.21 mV, respectively (*p* < 0.001), and 7.13 ± 2.41 and 5.90 ± 2.24 mV, respectively (*p* < 0.001), for the left peroneal nerve. Reductions in the CMAP amplitude of the peroneal nerve were significantly lower in the right foot compared to the left foot (−9.58 vs. −16.8, *p* = 0.043). Application of the FS did not significantly decrease the overall incidence of skin and nail toxicity compared with the left foot during the study period (all *p* > 0.05). **Conclusions**: Docetaxel induced motor and sensory PN, but the use of a FS resulted in a smaller reduction in peroneal nerve amplification three months after the end of chemotherapy.

## 1. Introduction

Taxanes, such as docetaxel, paclitaxel, and nanoparticle albumin-bound paclitaxel, are the most commonly used cytotoxic agents for breast carcinoma [1]. They suppress the function of microtubules, which is important for cellular function [2]. Docetaxel, one of the most commonly used taxanes, induces toxicities such as myelosuppression, alopecia, peripheral neuropathy (PN), and skin and nail toxicity [3,4]. Skin and nail toxicities include erythematous changes of the skin, hand-foot syndrome (HFS), and nail and toe changes [5,6]. These changes persist temporarily but sometimes can last for years, and there are no known preventive strategies [7,8]. The likelihood of taxane-induced PN depends on the type of taxane used, dose, and schedule [9].

In previous studies, the use of a frozen sock (FS) or glove has been shown to decrease the incidence of docetaxel-induced hand and foot nail toxicities [10,11]. It can also decrease the symptoms of paclitaxel-induced PN [12]. Cryotherapy decreases regional perfusion by inducing vasoconstriction and decreasing uptake of the chemotherapeutic agent, causing less damage to neurons or mechanotransductions [13]. However, one limitation of previous studies is that they all used a subjective measurement, such as the Common Terminology Criteria for Adverse Events (CTCAE), Patient Neurotoxicity Questionnaire, or Functional Assessment of Cancer Therapy, as an endpoint, and these results could be biased by placebo effects [12,14,15,16,17]. Only one study by Bandla et al. provided an objective evaluation of chemotherapy-induced peripheral neuropathy (CIPN) using nerve conduction study (NCS) [18]. Therefore, the first aim of this study was to evaluate the role of FS treatment in preventing docetaxel-induced PN as assessed by NCS. The secondary aim was to evaluate the subjective symptom scale of HFS symptoms, quality of life (QoL), and global discomfort based on a subjective tool using CTCAE.

## 2. Materials and Methods

### 2.1. Patients

This single-institution prospective study (Clinical Research Information Service, KCT0002662) was conducted from October 2017 to October 2018 and approved by the Institutional Review Board of Kangbuk Samsung Hospital (Approval No. 2017-05-020). Informed consent was obtained from all patients before initiation of the study. Patients were eligible if they were 20–75 years old; had American Joint Committee on Cancer TNM stage T0–T4, N1–N2, or M0; had an Eastern Cooperative Oncology Group Performance status of 0 or 1; and planned for adjuvant or neoadjuvant chemotherapy with docetaxel administration at a dose of 75–100 mg/m^2^ via a 90 min intravenous infusion every three weeks, alone or in combination with other cytotoxic agents. Patients were excluded if they had prior treatment with chemotherapeutic agents, including taxanes or platinum agents; skin or nail disorders; a history of PN (grade 2 or higher); Raynaud syndrome; peripheral vascular disease; or cold intolerance.

### 2.2. Cold Therapy

Patients wore an Elasto-Gel (84400 APT Cedex, Akromed, Saignon, France) flexible FS, which was the same product used in a previous study [10]. This patented sock contained glycerin whose thermal properties enable it to be used in hot or cold therapies. The gel-filled FS covered the foot to the ankle. Before use, it was refrigerated for at least 3 h at −10 to −20 °C. Similar to Scotté et al. study of FS, each patient wore a FS for a total of 90 min on the right foot (15 min before the administration of docetaxel, 1 h during the docetaxel infusion, and 15 min after the end of the infusion) with every docetaxel infusion [10]. Three FSs were used successively (for 30 min each) in this study to maintain a consistently low temperature of the foot. To confirm the maintenance of the low temperature and the safety of the FS, we used a thermoregulator attached to the FS to measure the temperature of the FS in ambient air after it was initially taken out of the refrigerator and repeated the temperature measurement after 5, 15, and 30 min. This same set of measurements was performed when the FS was removed from the refrigerator and placed on the foot (Figures S1 and S2). The study was prematurely stopped if the patients experienced cold intolerance and discomfort during the FS application, pain during NCS, had a serious adverse event, or withdrew consent. The study was not blinded. The left foot was not protected by the FS and served as a control.

### 2.3. Assessment of Neuropathy

Motor and sensory NCS were performed before the first cycle of docetaxel (NCSpre) and three months after the end of the last cycle (NCS3mo) on both feet. Sensory nerve action potential (SNAP) amplitudes and nerve conduction velocities (NCVs) were measured in the bilateral sural, superficial peroneal, and medial plantar nerves [19]. Compound motor action potential (CMAP) amplitudes and motor NCVs were measured in the bilateral common peroneal and tibial nerves [20].

### 2.4. Assessment of Skin and Nail Toxicities

Skin and nail toxicities were assessed at each cycle and three months after the last cycle of docetaxel infusion by a medical investigator (E.Y.K.) using the National Cancer Institute CTCAE (version 3) [21], and changes were documented photographically at each cycle.

The grades of skin disorders were classified as follows. Grade 1 indicates minimal skin changes or dermatitis (e.g., erythema, edema, or hyperkeratosis) without pain. Grade 2 indicates skin changes (e.g., peeling, blisters, bleeding, fissures, edema, or hyperkeratosis) with pain, limiting instrumental activities of daily living (ADL). Grade 3 includes severe skin changes (e.g., peeling, blisters, bleeding, fissures, edema, or hyperkeratosis) with pain, limiting self-care ADL.

The grades of nail disorder were classified as follows: grade 1, discoloration, ridging (koilonychia), or pitting; grade 2, partial loss of nail(s) (onycholysis) or pain in nail beds not interfering with function; and grade 3, partial loss of nail(s) (onycholysis) or pain in nail beds interfering with function or complete loss of nail(s).

### 2.5. Hand-Foot Syndrome Symptom and Quality of Life Questionnaire, Global Discomfort

In 2015, Anderson et al. [22] developed the hand-foot skin reaction and quality of life (HF-QoL) questionnaire, which simultaneously measured the extent of HFS symptoms and their effects on daily activities. In 2016, Nam et al. [23] developed an HF QoL-K instrument, which was a valid and reliable questionnaire for the measurement of the symptoms and QoL in Korean cancer patients suffering from HFS. We integrated the questionnaire from both the HF-QoL questionnaire and the HF QoL-K instrument, and the list of questions is summarized in Table S1. The questionnaire focused on symptoms of neuropathy and ADL that correlate with QoL [24]. The patients responded to each question using a five-point rating system: 0-none, 1-mild, 2-moderate that does not interfere with ADL, 3-moderate neuropathy that interferes with ADL, and 4-very severe. The questionnaire was given at each cycle and three months after the last cycle of docetaxel infusion.

The level of discomfort of the patients during the FS application was also assessed using a similar four-point rating system. Assessment of the patient’s overall discomfort included factors such as glove contact, temperature tolerance, and immobilization constraints.

### 2.6. Statistical Analysis

Categorical data are summarized using frequencies and percentages. Continuous variables are shown as mean ± standard deviation (SD). Sensory and motor nerve parameters of amplitude and velocity, both before the first cycle of docetaxel (NCSpre) and three months after the end of the last cycle (NCS3mo), were analyzed and averaged across the patients. A paired *t*-test was used to compare the NCSpre and NCS3mo of each patient. A paired *t*-test was also performed to compare the NCS values between the right (FS) foot and left (control) foot [18]. For evaluation of skin and nail toxicities, McNemar’s test was used to determine the differences in the grade of skin and nail toxicities before chemotherapy and three months after the end of chemotherapy for both the right (FS) foot and left (control) foot. For evaluation of HFS symptoms, a paired *t*-test was used to compare differences in mean values of symptoms over time between the right (FS) foot and left (control) foot. The values were depicted with a line graph with 95% confidence intervals. For assessment of QoL, a paired *t*-test was used to compare the difference between the mean values of symptoms at each cycle to that of cycle 1. To compare the discomfort during FS use, mean values were summarized using frequencies and percentages or mean ± SD. Any missing data were excluded from analysis. Statistical analyses were performed using R 4.3.2 version (R Foundation for Statistical Computing, Vienna, Austria). *p* < 0.05 was considered to be statistically significant.

## 3. Results

### 3.1. Baseline Characteristics

**Figure 1:**
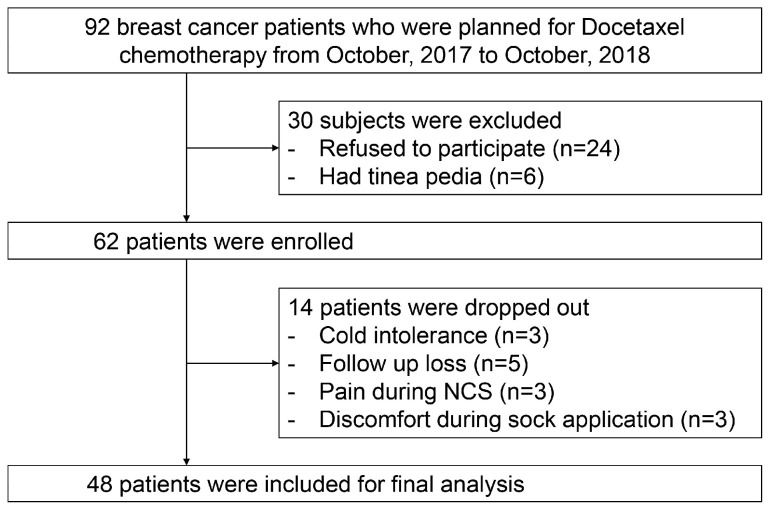
Flowchart of the selection process for the study participants.

Of the 92 patients who were planned for docetaxel chemotherapy from October 2017 to October 2018, 30 were excluded. As a result, a total of 62 patients were included in this study. During the study period, 14 patients were dropped due to cold intolerance (n = 3), follow-up loss (n = 5), pain during NCS (n = 3), and discomfort during FS application (n = 3). As a result, 48 patients were included in the final analysis (Figure 1).

The mean age at the time of enrollment was 49.8 years (range, 30–64 years). Docetaxel was administered either as monotherapy (39.5%) or in combination with trastuzumab, carboplatin, cyclophosphamide, or pertuzumab (60.5%). The characteristics of the patients are described in Table 1.

### 3.2. Nerve Conduction Study

NCS analyses were performed on both feet of the 48 subjects (Table 2). Motor NCS3mo was compared with NCSpre using a paired *t*-test. At baseline, the CMAPs of the peroneal and tibial nerve were not different between the right (FS) foot and left (control) foot (peroneal nerve, 7.6 ± 2.4 mV vs. 7.1 ± 2.4 mV, *p* > 0.05; tibial nerve, 27.9 ± 6.5 mV vs. 27.1 ± 6.1 mV, all *p* > 0.05). The NCS3mo analysis showed a decrease in the NCV of the peroneal nerve and the CMAP and NCV of the tibial nerve after three months compared to the baseline. The magnitude of decreases in NCV of the peroneal nerve, CMAP, and NCV of tibial nerve for both the right (FS) foot and the left (control) foot were not significantly different between the baseline and the three-month follow-up (all *p* > 0.05) except for the CMAP of the peroneal nerve. The CMAP amplitudes of the peroneal nerve between the baseline and three-month follow-up were significantly lower in the right (FS) foot compared to the left (control) foot (−9.58 mV vs. −16.8 mV, *p* = 0.043).

The sensory NCS3mo follow-up was also compared with NCSpre using a paired *t*-test. At baseline, the SNAPs and the NCVs of the sural, superficial peroneal, and medial plantar nerves were not different between the right (FS) foot and the left (control) foot (all *p* > 0.05). SNAP and the NCVs amplitudes decreased after three months compared with the baseline in both right (FS) foot and left (control) foot. The magnitude of decreases in SNAPs and NCVs of sural, superficial peroneal, and medial plantar nerve for both the right (FS) foot and left (control) foot were not significantly different between the baseline and three-month follow-up (all *p* > 0.05). FS did not significantly change sensory nerve outcomes compared with the control foot.

The decrease in all parameters of NCS at three months, as described in Table 2, suggests that docetaxel-induced peripheral motor and sensory neuropathy starts even as early as three months. It also proves that the test was adequately performed, clarifying the group of patients with true neuropathy. We performed subgroup analysis according to the total cumulative dose of docetaxel (dose reduction ≤300 mg/m^2^ or not >300 mg/m^2^) and whether it was accompanied with doxorubicin. None of the subgroup analyses showed statistically significant results (Table S2).

### 3.3. Skin and Nail Toxicities

**Figure 2:**
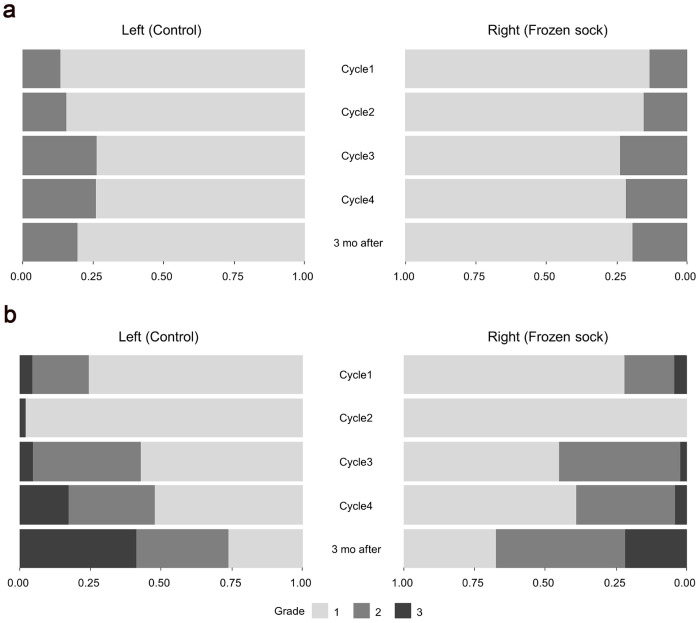
Assessment of (**a**) skin and (**b**) nail toxicity at each cycle and three months after the end of chemotherapy for both the right (FS) and left (control) foot.

Skin and nail toxicities are described in Table 3 and Figure 2. In total, nine patients (18.7%) experienced grade 1–2 skin toxicity in both the right (FS) and left (control) foot at three months after chemotherapy. Grade 1–2 nail toxicity was observed in the left (control) foot in 34 patients and in the right (FS) foot in 31 patients (64.6%) three months after chemotherapy. As the treatment cycles of docetaxel increased, the incidence of grade 1–2 nail toxicity also increased gradually for the left (control) foot. However, application of the FS did not significantly decrease the overall incidence of skin and nail toxicity over time (all *p* > 0.05). One patient showed grade 2 nail toxicity on the left (control) foot three months after chemotherapy (Figure S3).

### 3.4. Hand-Foot Syndrome Symptom

**Figure 3:**
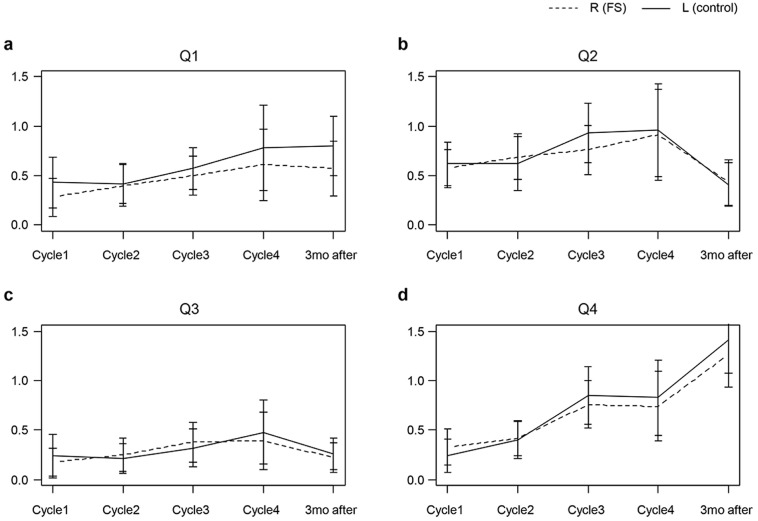

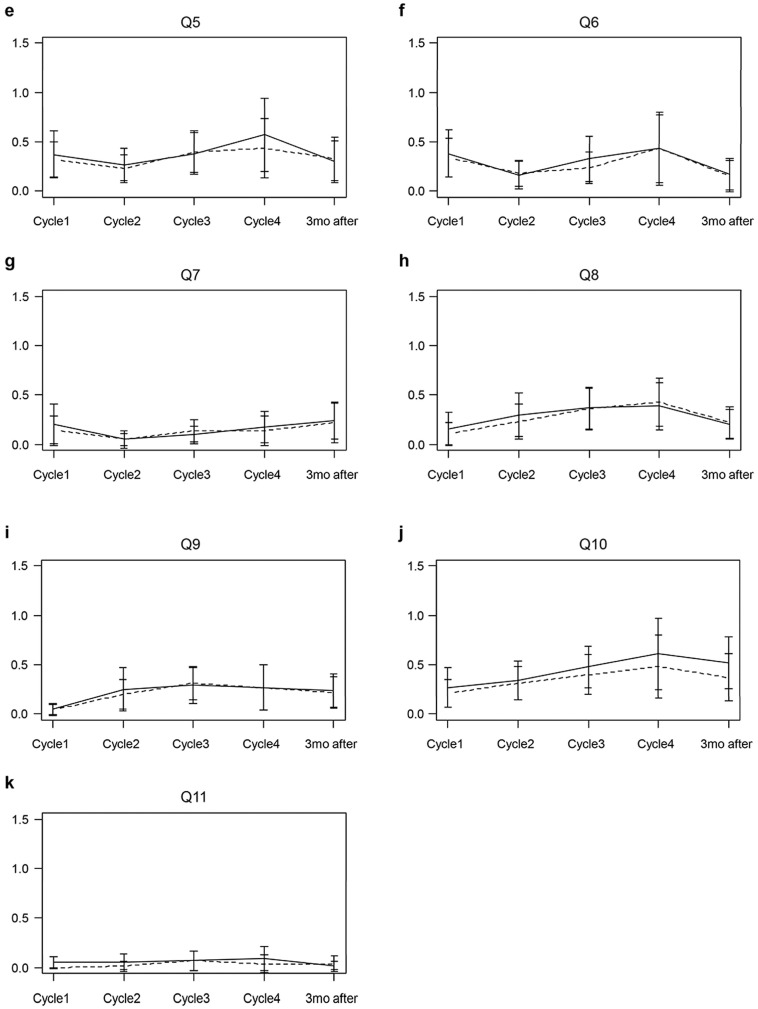
HFS questionnaire score at each cycle and three months after the end of chemotherapy between the right (FS) and left (control) foot. (**a**) Feel numbness or tingling; (**b**) Have no strength in foot; (**c**) Have red or discolored skin; (**d**) Show changes in color or appearance of toenails; (**e**) Painful; (**f**) Sensitive to pressure; (**g**) Burning or “hot” sensation; (**h**) Have cracked or peeling skin; (**i**) Have thickened or calloused skin; (**j**) Swollen; (**k**) Have blisters or sores. Dashed line; right (FS) foot, Solid line; left (control) foot.

HFS symptoms were recorded at each visit. The mean values and SDs of the HFS scales and the difference between the left and right feet are shown in Table 4 and Figure 3. Application of the FS was associated with a tendency toward a reduction of the mean score compared to the left foot per cycle. However, except for Q1 (feel numbness or tingling) and Q2 (have no strength at foot), there were no significant differences between the two feet in the mean score of the HFS. Three months after chemotherapy, the score for the left (control) foot was higher than that of the right (FS) foot with statistical significance for Q1 (0.80 ± 1.05 vs. 0.57 ± 0.96, *p* = 0.002). The difference was significant at the third cycle for Q2. For the left (control) foot, the score was 0.93 ± 1.00, while in the right (FS) foot, it was 0.76 ± 0.82 (*p* = 0.033). This indicated reduced symptoms in the foot treated with the FS.

### 3.5. Quality of Life Questionnaire

**Figure 4:**
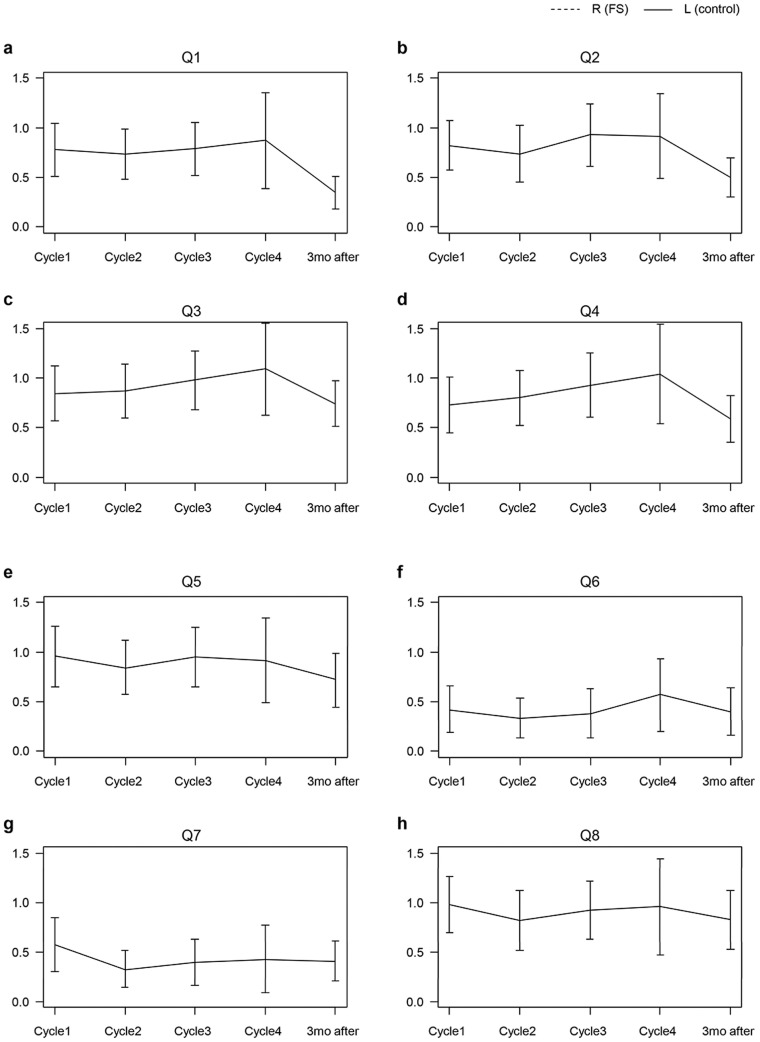

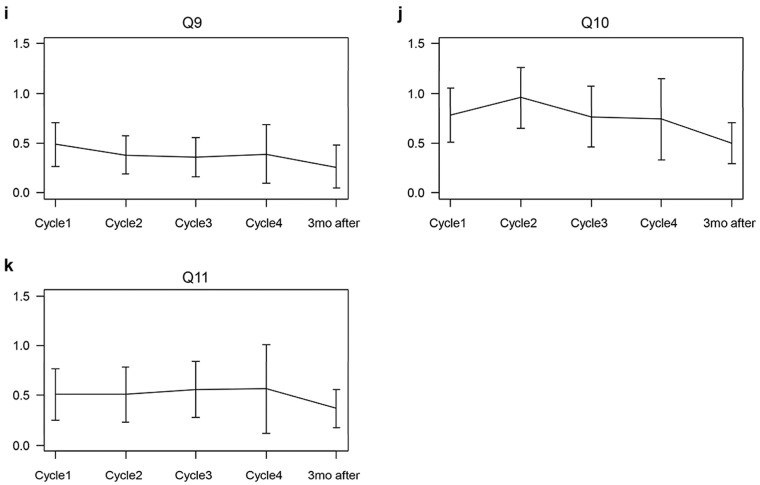
Assessment of QoL at each cycle and three months after the end of chemotherapy. (**a**) Avoid physical activity; (**b**) Difficulty doing heavy housework; (**c**) Walking at a slower pace; (**d**) Difficulty using stairs; (**e**) Difficulty lifting/carrying objects; (**f**) Have to wear different clothes/shoes; (**g**) Have to stay off feet; (**h**) Accomplish less than would like; (**i**) Difficulty bathing; (**j**) Less comfortable in social activities; (**k**) Feel isolated from others.

The score for the HF QoL-K represented the severity of symptoms of PN and, hence, the QoL of the patients (Table 5, Figure 4). Except for Q1, Q2, and Q7, all other questions did not show statistically significant differences between the first cycle and afterward. After receiving the second chemotherapy cycle, the QoL score was significantly decreased compared to that of the first cycle in Q7 (have to stay off feet, *p* = 0.031) and showed a decreased tendency at three months. After receiving the fourth chemotherapy cycle, the QoL score was significantly lower than that of the first cycle and at the three-month review for Q1 (avoid physical activity) and Q2 (difficulty doing heavy housework) (*p* = 0.028, *p* = 0.031, respectively). This indicated that patients still suffered deterioration from PN even after completing chemotherapy, which persisted for three months.

### 3.6. Discomfort During Frozen Sock Application

**Figure 5:**
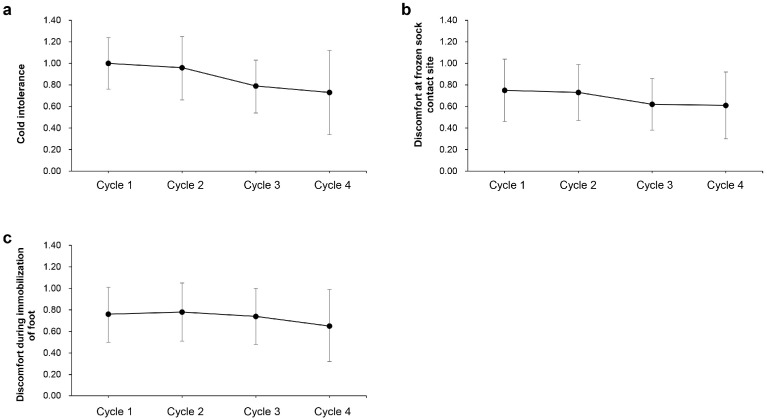
Assessment of discomfort at each cycle and three months after the end of chemotherapy. (**a**): Cold intolerance; (**b**): Discomfort at frozen sock contact site; (**c**): Discomfort during immobilization of foot.

Assessment of the discomfort of the patients included cold intolerance, FS contact site, and immobilization of the foot (Table 6, Figure 5). About 23% of the patients did not feel any discomfort from these three aspects during FS application, even during the fourth cycle of chemotherapy. Only two patients (4.2%) were dissatisfied because of severe cold intolerance in the fourth cycle. The mean score of each assessment decreased from first to last cycle, which indicated that the patient adapted to FS treatment over time.

## 4. Discussion

We found that the use of a FS resulted in a smaller reduction in peroneal nerve amplification three months after the end of chemotherapy. CIPN is associated with decreased QoL, suboptimal dosing, and even discontinuation of the chemotherapeutic agent [25,26]. A recently updated American Society of Clinical Oncology guideline states that no agents are recommended for the prevention of CIPN [27]. It states that although the proof of benefit has not been established, cryo-compression therapy may, in part, prevent CIPN symptoms and appear to be reasonably safe. In the guideline, six trials evaluated cryotherapy for decreasing CIPN, and four showed a positive result while two showed negative results. However, all these studies are based on a subjective symptom scale. There was only one study that objectively identified CIPN using NCS, as we did in our study [18].

The role of cryotherapy was first introduced by Danish investigators [28]. Bandla et al. [18] observed that the efficacy of cryo-compression in the preservation of peroneal motor nerve function was better than in a control group. However, sensory nerve function changes were not found to be different across the groups in other studies [29,30]. Studies of cryotherapy for preventing taxane-induced skin and nail toxicities reported mixed results. Scotté et al. reported that onycholysis and skin toxicity were significantly lower in a frozen glove-protected hand compared with the control hand (*p* = 0.0001) [11]. McCarthy et al. reported that no significant differences were determined between hand conditions in terms of time to event, nor in terms of toxicity in the gloved and non-gloved hands [31]. Our study also showed that the application of FS treatment did not significantly decrease the overall incidence of skin and nail toxicity over time (all *p* > 0.05). The negative result could be due to a suboptimal amount of docetaxel exposure because the severity of skin and nail toxicity is correlated with amount of exposure to docetaxel [32]. Ishiguro et al. published that none of the patients developed hand or nail toxicities after five months of frozen glove use [33].

There was no difference between the right (FS) and left (control) foot in the extent of the decrease in the motor and sensory nerve after three months compared to the baseline except for peroneal nerve. The decrease in all NCS parameters at three months indicated that docetaxel-induced peripheral motor and sensory neuropathy starts as early as three months. CIPN presents as nerve damage in a length-dependent pattern, and because the peroneal nerve is the longest motor nerve in a peripheral limb, it is a commonly used parameter to evaluate motor nerve function [34]. Motor NCS stimulates the proximal part of the peripheral nerve with supramaximal stimuli, and then the CMAP is recorded [35]. The amplitude of the CMAP indicates axonal damage, and the velocity indicates demyelination. In line with our findings, sensory nerve function changes after cryotherapy were not found to be different across studies [29,30,36]. This difference could be explained by the differential response of mitochondrial abnormality in axons after CIPN. In preclinical study, Xiao et al. found sensory dysfunction, but not motor dysfunction, in the rat after paclitaxel exposure [37]. They speculated that this may be due to a mitotoxic effect resulting from the primary afferent neuron’s cell body being exposed to high and persistent levels of paclitaxel. Thus, hypothetically, because of this toxic effect on mitochondria, even cryotherapy could not prevent sensory nerve dysfunction after CIPN.

This is the only study that objectively evaluated CIPN using NCS and was matched with a subjective symptom scale. We found that subjective HFS and QoL symptom scales were discordant with the results of the objective NCS test. In our study, SNAP and NCV amplitudes significantly decreased after three months compared to the baseline in both right (FS) foot and left (control) foot. However, the score for HFS questionnaire decreased until the fourth cycle and then rose again three months after chemotherapy. The reason for the discordance is possibly due to non-masked study design, which could lead to bias in the patient-reported outcome. This further indicated that we need more objective scale assessments with long-term follow-up for CIPN.

In our study, only three patients dropped out due to cold intolerance, which indicated that most patients could tolerate the cold, although clinicians and patients should still be aware of frostbite risk. In previous trials, a significant portion of patients dropped out due to cold intolerance [11,15]. Another study also assessed the tolerability of the cooling therapy using validated scales such as a visual analogue pain scale, a subjective tolerance scale, and a shivering assessment scale [18]. The reason for the low level of cold intolerability in our study was probably due to maintaining the temperature of FS in the upper normal range. Ishiguro et al. showed that a frozen glove prepared at −10 °C was as effective at preventing CIPN gloves at −25 or −30 °C [33]. Chitkumarn et al. insisted that maintaining the surface temperature of the frozen glove at 10–20 °C was effective for preventing CIPN [38]. We also measured the temperature of the FS both on and off the foot when it was first removed from the refrigerator and again after 5, 15, and 30 min (Figures S1 and S2). In the future, a detailed safety protocol is needed to reduce cold intolerance and to provide early detection for patients who are intolerable to FS.

The present study had some limitations. First, the study sample comprised only participants who underwent docetaxel chemotherapy, and the responses might have been different with other chemotherapeutic agents and different durations, such as with paclitaxel. Second, because the study was not blinded to both patients and examiners, a bias could have impacted the patient-reported outcome. Third, the study was comprised of a small number (only 48 patients) and had no data on the influence of other chemotherapeutic agents on PN and effect of FS on CMPA amplitude in these patients. Finally, the follow-up period was short, and we needed follow-up longer than three months to discover any long-lasting protective effect of FS.

## 5. Conclusions

We found that reductions in the CMAP amplitude of the peroneal nerve were significantly lower in the FS-protected right foot compared to the left foot three months after the completion of docetaxel chemotherapy, as determined with NCS. Because no effective prevention tools are currently available for preventing CIPN, FS can be applied to patients who are undergoing chemotherapy.

## Figures and Tables

**Table 1:** Patient clinicopathological characteristics.

Characteristics	Value
Age (years)	49.81 ± 8.62
Menopausal status	
Premenopausal	29 (60.4)
Postmenopausal	19 (39.6)
AJCC TNM stage	
I	11 (22.9)
II	28 (58.3)
III	9 (18.8)
Chemotherapeutic regimen	
(A) AC + T	10 (20.8)
(A) AC + TH	1 (2.1)
(A) TC	22 (45.9)
(A) TCH	1 (2.1)
(N) AC + T	10 (20.8)
(N) TCHP	4 (8.3)
Docetaxel	
Single agent	19 (39.5)
Combination therapy	29 (60.5)
No. of cycles	4.21 ± 0.62
Cumulative docetaxel dose, mg	400 (300–450)

AJCC, American Joint Committee on Cancer; A, adjuvant; AC, adriamycin/cyclophosphamide; T, docetaxel; TH, docetaxel/trastuzumab; TC, docetaxel/cyclophosphamide; TCH, docetaxel/carboplatin/trastuzumab; N, neoadjuvant; TCHP, docetaxel/carboplatin/trastuzumab/pertuzumab. Values are presented as numbers (%) mean ± standard deviation.

**Table 2:** Nerve conduction study at the baseline (NCS pre) and three months after the end of chemotherapy (NCS 3mo).

Motor Nerve	Left (Control)				Right (Frozen Sock)				*p*-Value (R vs. L)
	NCS pre	NCS 3mo	*p*-Value	Δ	NCS pre	NCS 3mo	*p*-Value	δ	
Peroneal nerve									
CMAP	7.13 ± 2.41	5.90 ± 2.24	<0.001	−16.8 (−1.23)	7.64 ± 2.42	6.81 ± 2.21	<0.001	−9.58 (−0.82)	0.043
NCV	49.88 ± 3.22	48.85 ± 3.01	0.005	−1.93 (−1.03)	50.06 ± 3.44	49.19 ± 2.93	0.009	−1.6 (−0.88)	0.704
Tibial nerve									
CMAP	27.1 ± 6.15	24.28 ± 5.38	<0.001	−9.43 (−2.82)	27.94 ± 6.47	25.41 ± 6.20	<0.001	−8.69 (−2.52)	0.626
NCV	50.4 ± 3.31	49.62 ± 2.88	0.080	−1.3 (−0.78)	50.44 ± 3.24	49.48 ± 3.02	0.030	−1.7 (−0.96)	0.633
Sural nerve									
SNAP	25.78 ± 10.43	18.71 ± 7.60	<0.001	−20.77 (−7.07)	26.40 ± 11.39	19.90 ± 8.18	<0.001	−14.39 (−6.5)	0.405
NCV	46.27 ± 1.95	43.82 ± 3.38	<0.001	−5.27 (−2.45)	46.09 ± 2.73	43.81 ± 3.50	<0.001	−4.82 (−2.28)	0.538
Superficial peroneal									
SNAP	22.20 ± 9.17	14.79 ± 6.42	<0.001	−30.14 (−7.41)	21.86 ± 8.09	15.30 ± 6.44	<0.001	−28.06 (−6.56)	0.355
NCV	42.51 ± 2.08	39.59 ± 3.14	<0.001	−6.84 (−2.92)	41.77 ± 2.80	39.23 ± 3.46	<0.001	−6.03 (−2.54)	0.241

CMAP, compound motor action potential; NCV, nerve conduction velocity; SNAP, sensory nerve action potential. Data are presented as mean ± standard deviation. Relative changes are shown as δ. Standard error of the mean is shown in parentheses. Relative change = (Value of indicator of NCS 3mo-Value of indicator of NCS pre)/Value of indicator of NCS pre × 100.

**Table 3:** Assessment of skin and nail toxicity at each cycle and three months after the end of chemotherapy (for both left and right feet).

	Number	Left (Control)		Right (Frozen Sock)		*p*-Value
		Grade 0	Grade 1–2	Grade 0	Grade 1–2	
Skin						
Cycle 1	45	39 (86.67)	6 (13.33)	39 (86.67)	6 (13.33)	>0.999
Cycle 2	45	38 (84.44)	7 (15.56)	38 (84.44)	7 (15.56)	>0.999
Cycle 3	42	31 (73.81)	11 (26.19)	32 (76.19)	10 (23.81)	>0.999
Cycle 4	23	17 (73.91)	6 (26.09)	18 (78.26)	5 (21.74)	>0.999
3 m after chemo	46	37 (80.43)	9 (19.57)	37 (80.43)	9 (19.57)	>0.999
Nail						
Cycle 1	45	34 (75.56)	11 (24.44)	35 (77.78)	10 (22.22)	>0.999
Cycle 2	45	44 (97.78)	1 (2.22)	45 (100)	0 (0)	>0.999
Cycle 3	42	24 (57.14)	18 (42.86)	23 (54.76)	19 (45.24)	>0.999
Cycle 4	23	12 (52.17)	11 (47.83)	14 (60.87)	9 (39.13)	0.480
3 m after chemo	46	12 (26.09)	34 (73.91)	15 (32.61)	31 (67.39)	0.248

Data are presented as numbers (%).

**Table 4:** Assessment of hand-foot syndrome symptoms at each cycle and three months after the end of chemotherapy.

	Number	Left (Control)		Right (Frozen Sock)		Mean Difference Right-Left	*p*-Value
Q1							
Cycle 1	47	0.43 ± 0.9	(0.17–0.69)	0.28 ± 0.65	(0.09–0.47)	−0.15 ± 0.66	0.128
Cycle 2	45	0.42 ± 0.69	(0.22–0.62)	0.40 ± 0.72	(0.19–0.61)	−0.02 ± 0.45	0.743
Cycle 3	42	0.57 ± 0.7	(0.36–0.78)	0.50 ± 0.67	(0.30–0.70)	−0.07 ± 0.51	0.372
Cycle 4	23	0.78 ± 1.04	(0.35–1.21)	0.61 ± 0.89	(0.25–0.97)	−0.17 ± 0.49	0.103
Three months after chemo	46	0.80 ± 1.05	(0.50–1.10)	0.57 ± 0.96	(0.29–0.85)	−0.24 ± 0.48	0.002
Q2							
Cycle 1	47	0.62 ± 0.77	(0.40–0.84)	0.57 ± 0.68	(0.38–0.76)	−0.04 ± 0.46	0.533
Cycle 2	45	0.62 ± 0.91	(0.35–0.89)	0.69 ± 0.79	(0.46–0.92)	0.07 ± 0.50	0.372
Cycle 3	42	0.93 ± 1.00	(0.63–1.23)	0.76 ± 0.82	(0.51–1.01)	−0.17 ± 0.49	0.033
Cycle 4	23	0.96 ± 1.15	(0.49–1.43)	0.91 ± 1.12	(0.45–1.37)	−0.04 ± 0.21	0.328
Three months after chemo	46	0.41 ± 0.75	(0.19–0.63)	0.43 ± 0.78	(0.20–0.66)	0.02 ± 0.33	0.660
Q3							
Cycle 1	45	0.24 ± 0.74	(0.02–0.46)	0.18 ± 0.49	(0.04–0.32)	−0.07 ± 0.50	0.372
Cycle 2	43	0.21 ± 0.51	(0.06–0.36)	0.25 ± 0.58	(0.08–0.42)	0.05 ± 0.49	0.533
Cycle 3	41	0.32 ± 0.61	(0.13–0.51)	0.38 ± 0.66	(0.18–0.58)	0.07 ± 0.41	0.262
Cycle 4	23	0.48 ± 0.79	(0.16–0.80)	0.39 ± 0.72	(0.10–0.68)	−0.09 ± 0.29	0.162
Three months after chemo	46	0.26 ± 0.57	(0.10–0.42)	0.22 ± 0.52	(0.07–0.37)	−0.02 ± 0.45	0.743
Q4							
Cycle 1	45	0.24 ± 0.57	(0.07–0.41)	0.33 ± 0.63	(0.15–0.51)	0.09 ± 0.51	0.253
Cycle 2	43	0.40 ± 0.62	(0.21–0.59)	0.42 ± 0.59	(0.24–0.60)	0.02 ± 0.27	0.570
Cycle 3	41	0.85 ± 0.94	(0.56–1.14)	0.76 ± 0.80	(0.52–1.00)	−0.10 ± 0.49	0.210
Cycle 4	23	0.83 ± 0.94	(0.45–1.21)	0.74 ± 0.86	(0.39–1.09)	−0.09 ± 0.42	0.328
Three months after chemo	46	1.41 ± 1.15	(1.08–1.74)	1.26 ± 1.10	(0.94–1.58)	−0.15 ± 0.63	0.109
Q5							
Cycle 1	46	0.37 ± 0.83	(0.13–0.61)	0.32 ± 0.63	(0.14–0.50)	−0.07 ± 0.57	0.445
Cycle 2	44	0.27 ± 0.54	(0.11–0.43)	0.23 ± 0.48	(0.09–0.37)	−0.05 ± 0.21	0.160
Cycle 3	42	0.38 ± 0.70	(0.17–0.59)	0.40 ± 0.70	(0.19–0.61)	0.02 ± 0.60	0.800
Cycle 4	23	0.57 ± 0.90	(0.20–0.94)	0.43 ± 0.73	(0.13–0.73)	−0.13 ± 0.55	0.266
Three months after chemo	46	0.30 ± 0.73	(0.09–0.51)	0.33 ± 0.76	(0.11–0.55)	0.02 ± 0.45	0.743
Q6							
Cycle 1	45	0.38 ± 0.81	(0.14–0.62)	0.34 ± 0.67	(0.14–0.54)	−0.04 ± 0.52	0.570
Cycle 2	44	0.16 ± 0.48	(0.02–0.30)	0.18 ± 0.45	(0.05–0.31)	0.02 ± 0.27	0.570
Cycle 3	42	0.33 ± 0.75	(0.10–0.56)	0.24 ± 0.53	(0.08–0.40)	−0.10 ± 0.69	0.377
Cycle 4	23	0.43 ± 0.90	(0.06–0.80)	0.43 ± 0.84	(0.09–0.77)	0 ± 0.30	>0.999
Three months after chemo	46	0.17 ± 0.57	(0.01–0.33)	0.15 ± 0.56	(0–0.31)	−0.02 ± 0.26	0.569
Q7							
Cycle 1	44	0.20 ± 0.70	(0–0.41)	0.15 ± 0.47	(0.01–0.29)	−0.05 ± 0.68	0.660
Cycle 2	44	0.05 ± 0.30	(0–0.14)	0.05 ± 0.21	(0–0.11)	0 ± 0.38	>0.999
Cycle 3	41	0.10 ± 0.30	(0.01–0.19)	0.14 ± 0.35	(0.03–0.25)	0.05 ± 0.22	0.160
Cycle 4	22	0.18 ± 0.39	(0.02–0.34)	0.14 ± 0.35	(0–0.29)	−0.05 ± 0.21	0.329
Three months after chemo	46	0.24 ± 0.67	(0.05–0.43)	0.22 ± 0.70	(0.02–0.42)	−0.02 ± 0.26	0.569
Q8							
Cycle 1	44	0.16 ± 0.57	(0–0.33)	0.11 ± 0.38	(0–0.22)	−0.05 ± 0.30	0.323
Cycle 2	44	0.30 ± 0.73	(0.08–0.52)	0.23 ± 0.60	(0.05–0.41)	−0.07 ± 0.33	0.183
Cycle 3	41	0.37 ± 0.70	(0.16–0.58)	0.36 ± 0.69	(0.15–0.57)	0 ± 0.45	>0.999
Cycle 4	23	0.39 ± 0.58	(0.15–0.63)	0.43 ± 0.59	(0.19–0.67)	0.04 ± 0.21	0.328
Three months after chemo	45	0.20 ± 0.50	(0.05–0.35)	0.22 ± 0.55	(0.06–0.38)	−0.02 ± 0.50	0.767
Q9							
Cycle 1	44	0.05 ± 0.21	(0–0.11)	0.05 ± 0.21	(0–0.10)	0 ± 0	-
Cycle 2	44	0.25 ± 0.75	(0.03–0.47)	0.20 ± 0.50	(0.05–0.35)	−0.07 ± 0.45	0.323
Cycle 3	41	0.29 ± 0.60	(0.11–0.47)	0.31 ± 0.56	(0.14–0.48)	0.02 ± 0.47	0.743
Cycle 4	22	0.27 ± 0.55	(0.04–0.50)	0.27 ± 0.55	(0.04–0.50)	0 ± 0	-
Three months after chemo	46	0.24 ± 0.60	(0.07–0.41)	0.22 ± 0.56	(0.06–0.38)	−0.02 ± 0.15	0.323
Q10							
Cycle 1	44	0.27 ± 0.69	(0.07–0.47)	0.21 ± 0.46	(0.07–0.35)	−0.05 ± 0.53	0.570
Cycle 2	44	0.34 ± 0.68	(0.14–0.54)	0.31 ± 0.56	(0.14–0.48)	−0.05 ± 0.30	0.323
Cycle 3	42	0.48 ± 0.71	(0.27–0.69)	0.40 ± 0.66	(0.20–0.60)	−0.07 ± 0.34	0.183
Cycle 4	23	0.61 ± 0.89	(0.25–0.97)	0.48 ± 0.79	(0.16–0.80)	−0.13 ± 0.34	0.083
Three months after chemo	46	0.52 ± 0.89	(0.26–0.78)	0.37 ± 0.83	(0.13–0.61)	−0.15 ± 0.56	0.070
Q11							
Cycle 1	44	0.05 ± 0.21	(0–0.11)	0 ± 0	(0–0)	−0.05 ± 0.21	0.160
Cycle 2	44	0.05 ± 0.30	(0–0.14)	0.02 ± 0.15	(0–0.06)	−0.02 ± 0.35	0.660
Cycle 3	42	0.07 ± 0.34	(0–0.17)	0.07 ± 0.34	(0–0.17)	0 ± 0	-
Cycle 4	23	0.09 ± 0.29	(0–0.21)	0.04 ± 0.21	(0–0.13)	−0.04 ± 0.21	0.328
Three months after chemo	46	0.02 ± 0.15	(0–0.06)	0.04 ± 0.29	(0–0.12)	0.02 ± 0.15	0.323

Data are presented as mean ± standard deviation (95% CI). Mean difference = mean value of left foot-mean value of right foot.

**Table 5:** Assessment of QoL at each cycle and three months after the end of chemotherapy.

	Cycle 1	Cycle 2	Cycle 3	Cycle 4	Three Months After Chemo
Q1					
Number	45	45	42	23	46
Mean ± SD	0.78 ± 0.90	0.73 ± 0.86	0.79 ± 0.87	0.87 ± 1.18	0.35 ± 0.57
95% CI	(0.51–1.04)	(0.48–0.99)	(0.52–1.05)	(0.39–1.35)	(0.18–0.51)
Mean difference (Cycle 1–each cycle)	-	0.12 ± 0.82	0.03 ± 0.92	−0.09 ± 1.08	0.45 ± 1.07
*p*-Value	-	>0.999	>0.999	>0.999	0.028
Q2					
Number	45	45	42	23	46
Mean ± SD	0.82 ± 0.86	0.73 ± 0.99	0.93 ± 1.05	0.91 ± 1.04	0.50 ± 0.69
95% CI	(0.57–1.07)	(0.45–1.02)	(0.61–1.24)	(0.49–1.34)	(0.30–0.70)
Mean difference (Cycle 1–each cycle)	-	0.21 ± 0.94	0.00 ± 1.06	−0.13 ± 0.97	0.36 ± 0.87
*p*-Value	-	0.607	>0.999	>0.999	0.031
Q3					
Number	45	45	42	23	46
Mean ± SD	0.84 ± 0.93	0.87 ± 0.92	0.98 ± 0.98	1.09 ± 1.12	0.74 ± 0.80
95% CI	(0.57–1.12)	(0.60–1.14)	(0.68–1.27)	(0.63–1.55)	(0.51–0.97)
Mean difference (Cycle 1–each cycle)	-	0.05 ± 0.65	−0.07 ± 0.76	−0.22 ± 0.95	0.16 ± 1.10
*p*-Value	-	>0.999	>0.999	>0.999	>0.999
Q4					
Number	45	45	42	23	46
Mean ± SD	0.73 ± 0.96	0.80 ± 0.97	0.93 ± 1.07	1.04 ± 1.22	0.59 ± 0.80
95% CI	(0.45–1.01)	(0.52–1.08)	(0.61–1.25)	(0.54–1.54)	(0.35–0.82)
Mean difference (Cycle 1–each cycle)	-	−0.07 ± 0.91	−0.17 ± 1.01	−0.26 ± 1.05	0.23 ± 1.03
*p*-Value	-	>0.999	>0.999	0.991	0.604
Q5					
Number	45	45	42	23	46
Mean ± SD	0.96 ± 1.04	0.84 ± 0.95	0.95 ± 0.99	0.91 ± 1.04	0.72 ± 0.96
95% CI	(0.65–1.26)	(0.57–1.12)	(0.65–1.25)	(0.49–1.34)	(0.44–0.99)
Mean difference (Cycle 1–each cycle)	-	0.14 ± 0.97	0.05 ± 0.88	0.22 ± 1.04	0.32 ± 1.18
*p*-Value	-	>0.999	>0.999	>0.999	0.320
Q6					
Number	45	45	42	23	45
Mean ± SD	0.42 ± 0.81	0.33 ± 0.71	0.38 ± 0.82	0.57 ± 0.90	0.40 ± 0.84
95% CI	(0.19–0.66)	(0.13–0.54)	(0.13–0.63)	(0.20–0.93)	(0.16–0.64)
Mean difference (Cycle 1–each cycle)	-	0.14 ± 0.83	0.07 ± 0.97	−0.22 ± 0.90	0.05 ± 1.00
*p*-Value	-	>0.999	>0.999	>0.999	>0.999
Q7					
Number	45	45	42	23	46
Mean ± SD	0.58 ± 0.92	0.33 ± 0.64	0.40 ± 0.77	0.43 ± 0.84	0.41 ± 0.72
95% CI	(0.31–0.85)	(0.15–0.52)	(0.17–0.64)	(0.09–0.78)	(0.21–0.62)
Mean difference (Cycle 1–each cycle)	-	0.30 ± 0.71	0.22 ± 0.86	0.17 ± 0.94	0.18 ± 0.90
*p*-Value	-	0.031	0.427	>0.999	0.742
Q8					
Number	45	45	42	24	46
Mean ± SD	0.98 ± 0.97	0.82 ± 1.03	0.93 ± 0.95	0.96 ± 1.20	0.83 ± 1.02
95% CI	(0.70–1.26)	(0.52–1.12)	(0.64–1.22)	(0.48–1.44)	(0.53–1.12)
Mean difference (Cycle 1–each cycle)	-	0.19 ± 1.10	0.10 ± 1.06	0.04 ± 1.15	0.18 ± 1.21
*p*-Value	-	>0.999	>0.999	>0.999	>0.999
Q9					
Number	45	45	42	23	46
Mean ± SD	0.49 ± 0.76	0.38 ± 0.65	0.36 ± 0.66	0.39 ± 0.72	0.26 ± 0.74
95% CI	(0.27–0.71)	(0.19–0.57)	(0.16–0.56)	(0.10- 0.69)	(0.05–0.48)
Mean difference (Cycle 1–each cycle)	-	0.14 ± 0.86	0.15 ± 0.80	0.09 ± 0.95	0.20± 1.05
*p*-Value	-	>0.999	0.977	>0.999	0.808
Q10					
Number	45	45	42	23	46
Mean ± SD	0.78 ± 0.93	0.96 ± 1.04	0.76 ± 1.01	0.74 ± 1.01	0.50 ± 0.72
95% CI	(0.51–1.05)	(0.65–1.26)	(0.46–1.07)	(0.33–1.15)	(0.29–0.71)
Mean difference (Cycle 1–each cycle)	-	−0.12 ± 1.14	0.15 ± 0.77	0.17 ± 1.03	0.30 ± 1.09
*p*-Value	-	>0.999	0.900	>0.999	0.318
Q11					
Number	45	45	41	23	46
Mean ± SD	0.51 ± 0.89	0.51 ± 0.97	0.56 ± 0.92	0.57 ± 1.08	0.37 ± 0.64
95% CI	(0.25–0.77)	(0.23–0.79)	(0.28–0.84)	(0.12–1.01)	(0.18–0.56)
Mean difference (Cycle 1–each cycle)	-	0.02 ± 1.14	−0.03 ± 0.87	−0.13 ± 1.22	0.16 ± 0.99
*p*-Value	-	>0.999	>0.999	>0.999	>0.999

CI, confidence interval; SD, standard deviation. Data are presented as mean ± standard deviation.

**Table 6:** Assessment of discomfort at each cycle and three months after the end of chemotherapy.

Comfort Assessment	Cold Intolerance	Discomfort at Frozen Sock Contact Site	Discomfort During Immobilization of Foot
Cycle 1			
Number	45	44	45
Mean ± SD	1.07 ± 0.84	0.75 ± 0.94	0.76 ± 0.86
95% CI	(1.82–2.31)	(1.47–2.03)	(1.51–2.01)
None	12 (25.53)	24 (51.06)	21 (44.68)
Mild	20 (42.55)	9 (19.15)	16 (34.04)
Moderate	11 (23.4)	9 (19.15)	6 (12.77)
Severe	2 (4.26)	2 (4.26)	2 (4.26)
N/A	2 (4.26)	3 (6.38)	2 (4.26)
Cycle 2			
Number	45	45	45
Mean ± SD	0.93 ± 0.94	0.73 ± 0.86	0.78 ± 0.90
95% CI	(1.66–2.21)	(1.48–1.99)	(1.51–2.04)
None	18 (38.30)	23 (48.94)	22 (46.81)
Mild	15 (31.91)	12 (25.53)	13 (27.66)
Moderate	9 (19.15)	9 (19.15)	8 (17.02)
Severe	3 (6.38)	1 (2.13)	2 (4.26)
N/A	2 (4.26)	2 (4.26)	2 (4.26)
Cycle 3			
Number	42	42	42
Mean ± SD	0.83 ± 0.85	0.62 ± 0.76	0.74 ± 0.83
95% CI	(1.58–2.09)	(1.39–1.85)	(1.49–1.99)
None	16 (34.04)	22 (46.81)	18 (38.30)
Mild	20 (42.55)	15 (31.91)	20 (42.55)
moderate	3 (6.38)	4 (8.51)	1 (2.13)
severe	3 (6.38)	1 (2.13)	3 (6.38)
N/A	5 (10.64)	5 (10.64)	5 (10.64)
Cycle 4			
Number	23	23	23
Mean ± SD	0.74 ± 0.86	0.61 ± 0.72	0.65 ± 0.78
95% CI	(1.39–2.09)	(1.31–1.90)	(1.34–1.97)
None	10 (21.28)	11 (23.40)	11 (23.40)
Mild	11 (23.4)	11 (23.40)	10 (21.28)
moderate	0 (0)	0 (0)	1 (2.13)
severe	2 (4.26)	1 (2.13)	1 (2.13)
N/A	24 (51.06)	24 (51.06)	24 (51.06)

CI, confidence interval; N/A, not available; SD, standard deviation. Data are presented as number (%) or mean ± standard deviation.

## Data Availability

The data presented in this study are available upon reasonable request from the corresponding author. The data are not publicly available due to ethical commitments for sensitive patient information.

## Supplementary Materials

The following supporting information can be downloaded at: https://www.mdpi.com/article/10.3390/jcm14030864/s1, Figure S1: Temperature of frozen sock without wearing FS; Figure S2: Temperature of frozen sock while wearing FS; Figure S3: Grade 2 nail toxicity on left (control) foot three months after chemotherapy; Table S1: Questionnaire form; Table S2: Subgroup analysis of nerve conduction study at the baseline and after three months.
