# Supplementary material for: The Use of Nerve Conduction Study to Evaluate the Effects of Frozen Sock Treatment on Docetaxel-Induced Peripheral Neuropathy in Breast Cancer Patients: A Prospective Clinical Trial
Source: J Clin Med. 2025 Jan 28;14(3):864. doi: 10.3390/jcm14030864 (PMC11818139; doi:10.3390/jcm14030864)
Supplement: Supplementary file 1 [file jcm-14-00864-s001.zip › jcm-3433948-supplementary.pdf]

Supplementary Materials

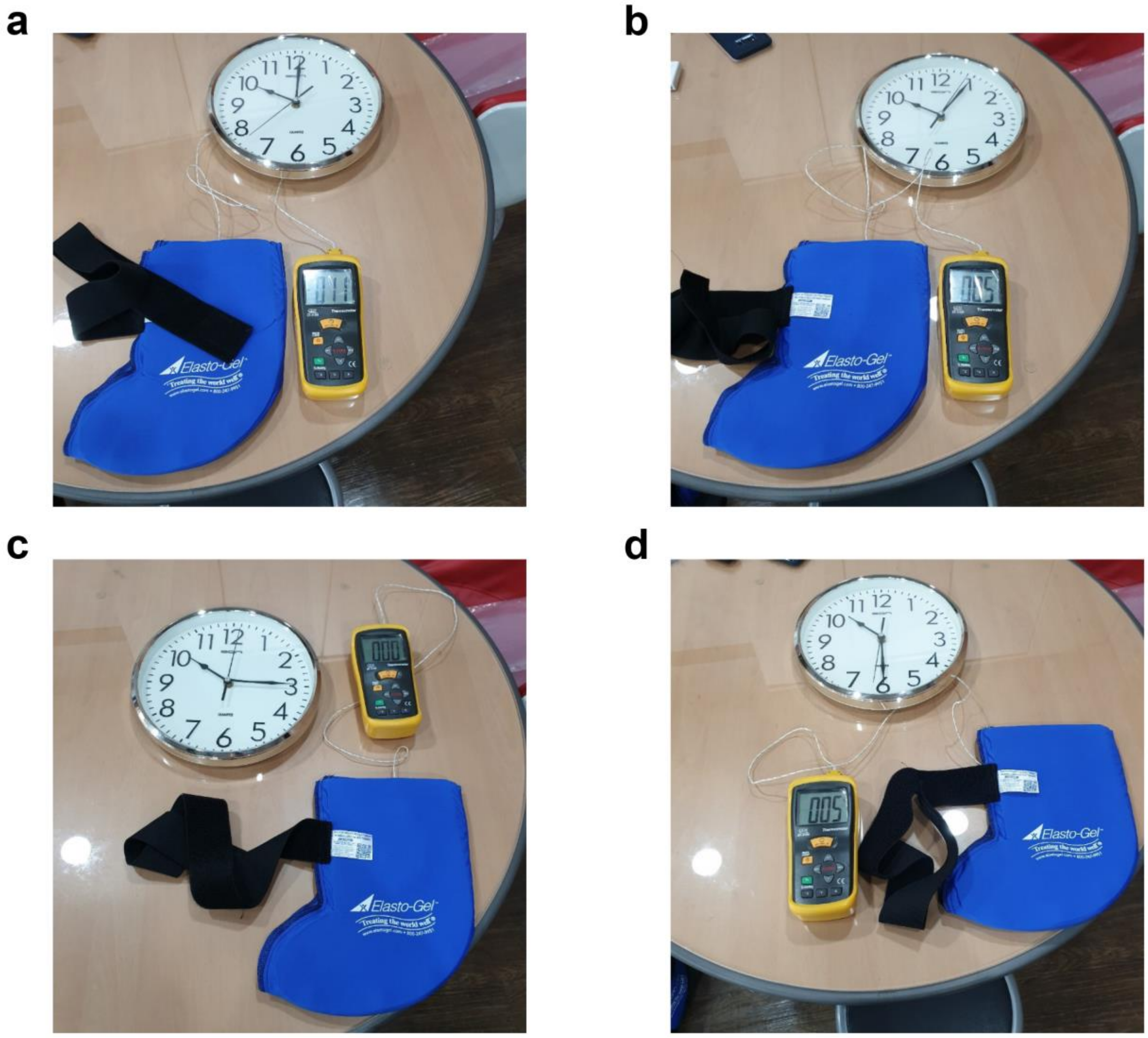

Figure S1. Temperature of frozen sock without wearing FS.

(a) at initial, (b) 5 minutes, (c) 15 minutes and (d) 30 minutes after taken out from refrigerator. The temperature was -11 °C, 5 °C, 0 °C, 5 °C respectively.

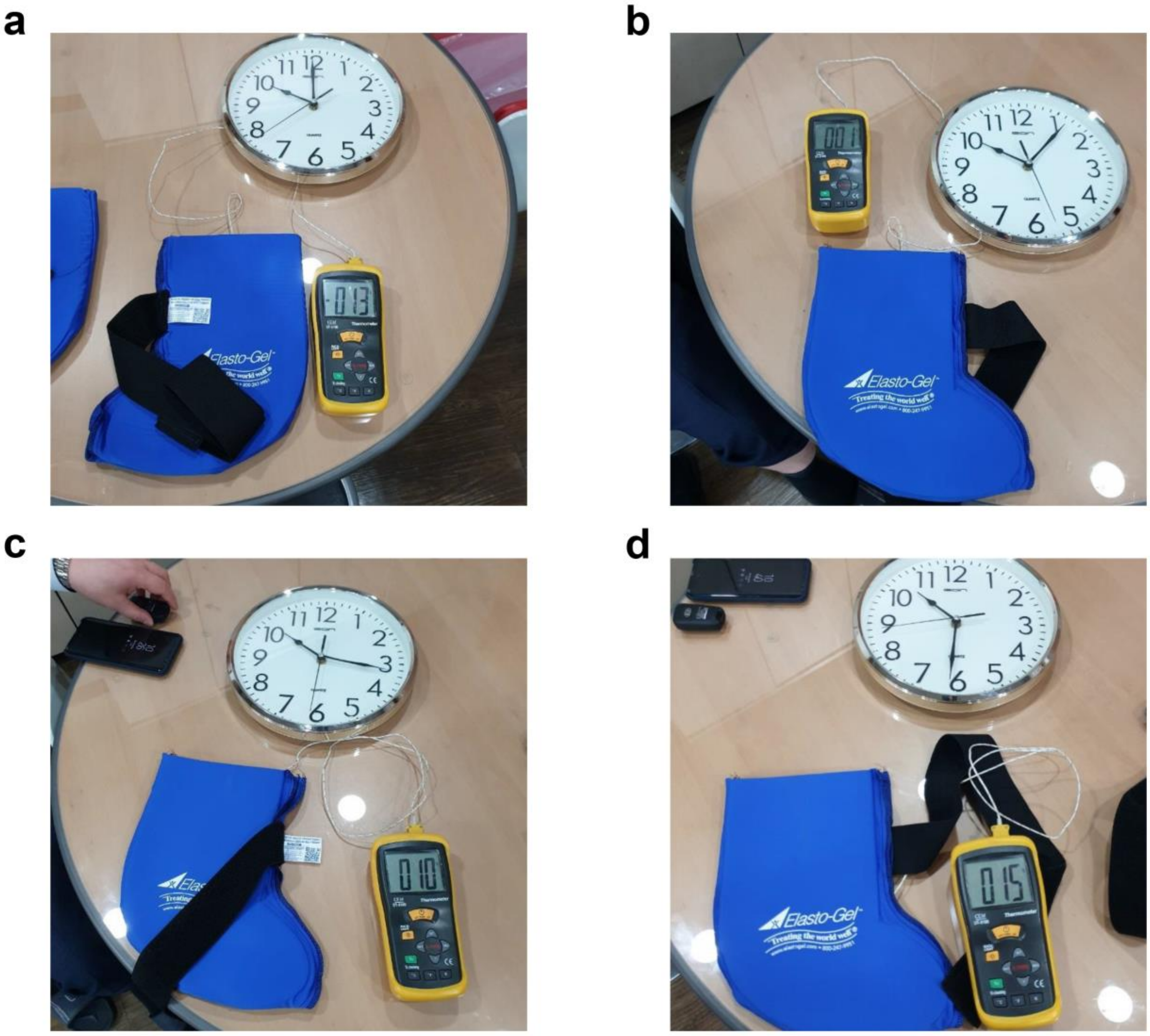

Figure S2. Temperature of frozen sock while wearing FS.

(a) at initial, (b) 5 minutes (c) 15 minutes and (d) 30 minutes after taken out from refrigerator. The temperature was -13°C, 1°C, 10°C, 15°C respectively.

a

b

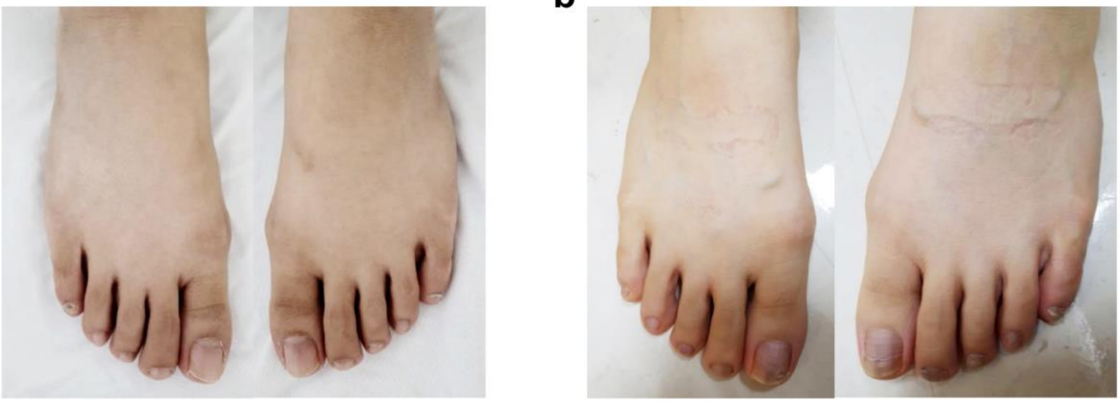

Figure S3. Grade 2 nail toxicity on left (control) foot three months after chemotherapy.

(a) Right and left foot (control) before 1st cycle of chemotherapy (b)Three months after last cycle of chemotherapy : Grade 2 nail toxicity (onycholysis) presented on the left (control) foot but absent on the right (FS) foot.

Table S1. Questionnaire form.

Hand-foot syndrome symptom					
Feet	1	2	3	4	5
Q1. Feel numbness or tingling					
Q2. Have no strength in foot					
Q3. Have red or discolored skin					
Q4. Show changes in color or appearance of toenails					
Q5. Painful					
Q6. Sensitive to pressure					
Q7. Burning or "hot" sensation					
Q8. Have cracked or peeling skin					
Q9. Have thickened or calloused skin					
Q10. Swollen					
Q11. Have blisters or sores					

Quality of life					
	1	2	3	4	5
Q1. Avoid physical activity					
Q2. Difficulty doing heavy housework					
Q3.Walking at a slower pace					
Q4. Difficulty using stairs					
Q5. Difficulty lifting/carrying objects					
Q6. Have to wear different clothes/shoes					
Q7. Have to stay off feet					
Q8. Accomplish less than would like					
Q9. Difficulty bathing					
Q10. Less comfortable in social activities					
Q11. Feel isolated from others					

Discomfort during frozen sock application				
Right foot	1	2	3	4
Cold intolerance				
Discomfort at frozen sock contact site				
Discomfort during immobilization of foot				

1; none
2; mild
3; moderate
4; severe

Table S2. Subgroup analysis of nerve conduction study at the baseline and after three months.

A. Patients with cumulative dose of docetaxel ≤300 mg/m² (n=22)					
	Parameter	Docetaxel	Right	Left	*p*-Value
Motor nerve					
Peroneal nerve	CMAP (mV)	Before	8.3 ± 2.9	7.5 ± 2.9	0.336
		After	7.5 ± 2.4	6.3 ± 2.4	0.115
	NCV (m/s)	Before	50.0 ± 3.5	49.8 ± 3.2	0.839
		After	49.6 ± 2.7	49.3 ± 2.9	0.621
Tibial nerve	CMAP (mV)	Before	29.0 ± 5.6	27.5 ± 5.9	0.325
		After	26.7 ± 6.0	26.7 ± 6.0	0.265
	NCV (m/s)	Before	49.9 ± 3.1	50.6 ± 3.2	0.405
		After	49.9 ± 2.0	50.0 ± 2.0	0.821
Sensory nerve					
Sural nerve	SNAP	Before	25.8 ± 11.0	24.9 ± 9.1	0.848
		After	22.8 ± 8.5	22.8 ± 8.5	0.573
	NCV	Before	46.2 ± 2.6	46.3 ± 1.7	0.796
		After	44.7 ± 2.9	44.7 ± 2.9	0.634
Superficial peroneal	SNAP	Before	22.6 ± 5.4	22.9 ± 6.7	0.886
		After	18.6 ± 5.0	18.2 ± 5.7	0.695
	NCV	Before	42.2 ± 2.1	43.1 ± 2.0	0.272
		After	40.4 ± 2.7	41.0 ± 2.4	0.576
Medial plantar	SNAP	Before	18.7 ± 5.0	17.9 ± 4.8	0.571
		After	15.4 ± 4.2	15.0 ± 4.4	0.779
	NCV	Before	47.5 ± 2.2	46.2 ± 3.6	0.227
		After	47.1 ± 3.0	46.2 ± 3.2	0.314

B. Patients with cumulative dose of docetaxel >300mg/m² (n=25)					
	Parameter	Docetaxel	Right	Left	*p*-Value
Motor nerve					
Peroneal nerve	CMAP (mV)	Before	7.1 ± 1.8	6.8 ± 2.0	0.540
		After	6.3 ± 1.9	5.6 ± 2.1	0.297
	NCV (m/s)	Before	49.7 ± 3.5	49.9 ± 3.3	0.931
		After	48.8 ± 3.1	48.5 ± 3.1	0.614
Tibial nerve	CMAP (mV)	Before	27.0 ± 7.1	26.8 ± 6.4	0.881
		After	24.3 ± 6.3	23.9 ± 5.3	0.577
	NCV (m/s)	Before	50.9 ± 3.4	50.2 ± 3.4	0.470
		After	49.1 ± 3.4	49.5 ± 3.3	0.768
Sensory nerve					
Sural nerve	SNAP	Before	26.9 ± 11.9	26.5 ± 11.6	0.924
		After	17.4 ± 7.1	16.3 ± 6.9	0.614
	NCV	Before	46.0 ± 2.9	46.2 ± 2.2	0.637
		After	43.0 ± 3.8	42.8 ± 3.2	0.767
Superficial peroneal	SNAP	Before	21.3 ± 10.0	21.6 ± 11.0	0.842
		After	12.4 ± 6.2	16.3 ± 6.9	0.686

	NCV	Before	41.4 ± 3.3	42.0 ± 2.0	0.491
		After	38.2 ± 3.7	38.4 ± 3.3	0.922
Medial plantar	SNAP	Before	16.7 ± 4.6	16.1 ± 5.2	0.487
		After	10.7 ± 4.1	10.0 ± 3.3	0.570
	NCV	Before	47.7 ± 3.2	47.4 ± 3.5	0.679
		After	44.8 ± 5.0	44.0 ± 4.4	0.422

C. Patients with treatment not accompanied by doxorubicin (n = 20)					
	Parameter	Docetaxel	Right	Left	*p*-Value
Motor nerve					
Peroneal nerve	CMAP (mV)	Before	8.5 ± 2.6	7.8 ± 2.7	0.603
		After	7.5 ± 2.4	6.5 ± 2.3	0.217
	NCV (m/s)	Before	50.6 ± 3.3	50.2 ± 3.0	0.811
		After	49.7 ± 2.6	49.7 ± 3.0	0.338
Tibial nerve	CMAP (mV)	Before	28.9 ± 5.3	27.3 ± 5.6	0.987
		After	26.6 ± 5.6	25.2 ± 5.6	0.345
	NCV (m/s)	Before	50.7 ± 3.4	51.0 ± 3.4	0.547
		After	50.5 ± 2.3	50.1 ± 2.5	0.221
Sensory nerve					
Sural nerve	SNAP	Before	26.4 ± 10.3	25.8 ± 8.8	0.998
		After	22.8 ± 7.9	21.5 ± 6.9	0.711
	NCV	Before	46.4 ± 2.7	46.5 ± 1.7	0.604
		After	44.8 ± 3.1	44.9 ± 3.2	0.738
Superficial peroneal	SNAP	Before	22.4 ± 5.7	23.2 ± 6.4	0.678
		After	18.1 ± 5.5	17.7 ± 5.8	0.526
	NCV	Before	42.3 ± 2.0	42.9 ± 2.1	0.477
		After	40.4 ± 2.7	40.6 ± 2.8	0.845
Medial plantar	SNAP	Before	18.7 ± 4.8	18.0 ± 4.5	0.501
		After	14.8 ± 4.4	14.4 ± 4.3	0.704
	NCV	Before	47.8 ± 2.3	46.8 ± 3.6	0.567
		After	47.1 ± 3.1	46.4 ± 3.2	0.134

D. Patients with treatment accompanied by doxorubicin (n=27)					
	Parameter	Docetaxel	Right	Left	*p*- Value
Motor nerve					
Peroneal nerve	CMAP (mV)	Before	6.5 ± 1.3	6.3 ± 1.6	0.723
		After	5.9 ± 1.6	5.0 ± 1.8	0.147
	NCV (m/s)	Before	49.4 ± 3.6	49.4 ± 3.4	0.799
		After	48.5 ± 3.3	47.8 ± 2.8	0.428
Tibial nerve	CMAP (mV)	Before	26.7 ± 7.7	26.8 ± 6.9	0.975

		After	23.9 ± 6.7	23.1 ± 5.0	0.546
	NCV (m/s)	Before	50.1 ± 3.1	49.6 ± 3.0	0.562
		After	48.1 ± 3.4	49.1 ± 3.3	0.320
Sensory nerve					
Sural nerve	SNAP	Before	26.4 ± 12.9	25.7 ± 12.5	0.955
		After	16.2 ± 7.2	15.2 ± 7.1	0.704
	NCV	Before	45.7 ± 2.7	45.9 ± 2.1	0.701
		After	42.5 ± 3.6	42.4 ± 3.1	0.837
Superficial peroneal	SNAP	Before	21.2 ± 10.5	21.0 ± 11.8	0.867
		After	11.8 ± 5.9	11.1 ± 5.3	0.625
	NCV	Before	41.2 ± 3.5	37.8 ± 3.8	0.486
		After	37.8 ± 3.8	38.3 ± 3.1	0.856
Medial plantar	SNAP	Before	16.3 ± 4.7	10.4 ± 3.9	0.407
		After	10.4 ± 3.9	9.5 ± 3.9	0.505
	NCV	Before	47.3 ± 3.3	44.2 ± 5.1	0.704
		After	44.2 ± 5.1	43.2 ± 4.3	0.428

Data are presented as median (range) or numbers (%).

CMAP, Compound motor action potential; NCV, nerve conduction velocity; SNAP, Sensory nerve action potential.
